# Overview about Oral Films in Mental Disorders

**DOI:** 10.3390/ph16081063

**Published:** 2023-07-26

**Authors:** Patrícia Batista, Manuela Pintado, Patrícia Oliveira-Silva

**Affiliations:** 1 Human Neurobehavioral Laboratory, Research Centre for Human Development, Universidade Católica Portuguesa, Rua Diogo Botelho, 1327, 4169-005 Porto, Portugal; 2 Laboratório Associado, CBQF—Centro de Biotecnologia e Química Fina, Escola Superior de Biotecnologia, Universidade Católica Portuguesa, Rua Diogo Botelho, 1327, 4169-005 Porto, Portugal

**Keywords:** oral films, mental disorders, delivery systems, psychotropic drugs

## Abstract

Mental disorders are increasing worldwide, and efforts have been developed by multidisciplinary research groups to combine knowledge from different areas such as psychology, neuroscience, medicine, and biotechnology to develop strategies and products to promote the prevention of mental disorders. Excessive antipsychotic consumption is a public health problem, and innovative strategies must be devised. The development of innovative and, if possible, natural products is one of the strategies to combat this public health problem. Oral films are recent delivery systems that have been developed with several advantages that should be applied in this area. This review intends to draw attention to these new dosage forms of drugs and bioactive molecules pertinent to the field of mental health prevention and therapy and to the need for regulatory guidelines to ensure their quality and safety. This is a critical overview about strengths, weaknesses, opportunities, and threats related to oral film implementation in mental disorder treatment.

## 1. Introduction

Mental disorders represent a big public health problem with an impact not only on health, but also a social and economic burden [1,2,3]. These clinical conditions account for more than 7% of the global burden of disease [2]. Commonly, these pathologies are treated with both pharmacotherapy and psychotherapy. Anxiolytic and hypnotic medications are some of the most prescribed in the majority of developed countries [3], while benzodiazepines are the psychotropic drugs most often consumed and prescribed. There are several types of these drugs: shorter-acting (mainly used as hypnotics), medium-acting, and long-acting (used as anxiolytics). However, these treatments are associated with significant adverse effects and social stigma toward the disease and its treatment [4,5].

These psychotropic drugs are usually consumed by oral administration as tablets. The oral route is the most commonly used because of its specific characterization and function in terms of permeability, drug bioavailability, avoidance of the degradation of the gastrointestinal tract, active local action and systemic administration, and acceptance by consumers [6,7]. Tablets, capsules, orodispersible tablets, drops, or syrup formulations are the most commonly used, but the pharmaceutical industry has been devising new drug dosage forms [6].

Oral disintegrating tablets represent a new dosage form, and they have been defined by the United States Food and Drug Administration as a solid drug dosage form with quick disintegration (30 s) [6]. Later came oral films, which are also called buccal films, orodispersible films, orally disintegrating films, sublingual films, mucoadhesive buccal film, or oral strips; these strips denote a new drug delivery system [8]. This system will be referred to as oral films (OF) throughout this paper. This delivery system can be characterized as a small and ultra-thin strip with active ingredients incorporated that quickly disintegrates once in contact with saliva in the oral cavity and can release the bioactive compounds or drugs incorporated into the strip [9].

OF presents several advantages, as will be addressed throughout the article. One is the ease of administration by risk groups, notably psychiatric patients. With this review, we intend to draw attention to these forms of drug or bioactive molecules in the field of mental health prevention and therapy.

## 2. Oral Film Characterization: Composition and Technologic Manufacturing

Oral film development needs to meet requirements in terms of aesthetics and performance, such as flavor, fast-dissolving properties, capacity to include and contain bioactive compounds, and physical appearance, among others. Furthermore, in terms of composition, all excipients used in the OF formulation should be Generally Regarded as Safe (GRAS) and need to have the drug formulation(s) approved.

**Figure 1:**
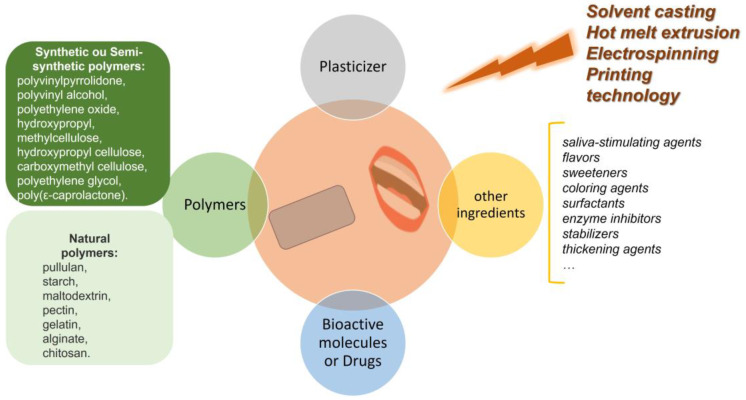
Schematic representation of the general composition of oral films.

The OF is composed of a polymeric matrix, usually made of hydrophilic polymers (Figure 1) [9]. Several polymers (natural, synthetic, or semi-synthetic) are known and available for use in the OF formulation, such as pectin, starch, hydroxy propyl cellulose, xanthan gum, guar gum, sodium alginate, chitosan, poly (ethylene oxide), and others [6,9,10,11]. Natural polymers obtained from natural sources are highlighted due to their properties such as non-toxicity, biocompatibility, and biodegradability. The composition can differ depending on the type of properties to be highlighted, for example, the mucoadhesion properties needed to ensure controlled release. Polymer selection is a challenge because of the condition of the OF’s properties (biological, chemical, and mechanical) and the methodology to be used in its production. Thus, the use of the correct polymer is often advantageous in terms of improving the performance of the delivery system [9].

In addition to polymers, another important compound in OF formulation is the plasticizer, which improves the OF flexibility. Glycerol, propylene glycol, and polyethylene glycol are some plasticizers commonly used. Thus, the selection of plasticizers will depend on the polymer, solvent, and drug or bioactive molecules’ compatibility.

The key ingredient is the bioactive molecule or drug to be delivered, which has a crucial role in the development of these delivery systems. However, the dosage that can be incorporated can be a limitation because it can alter the OF’s integrity. For example, OFs are unsuitable for administering high dosages [10].

The OF production is conditioned by not only the composition but also the process used during manufacturing. OF is commonly manufactured by solvent casting and semi-solid casting [12,13,14]. Hot melt extrusion, the electrospinning of drug-loaded polymeric solutions, and inkjet printing technology are the most recent forms of technology to be applied in the pharmaceutical area [8,9,10,11]. The type of processing may depend on the physical and chemical properties of the polymers used, as well as on the profile/delivery that is intended for the final product. Thus, selection of the polymers is an important phase for defining the processing methodology to ensure good processability, stability, and OF performance [10]. However, several parameters are considered during OF production: compounds, processability, biochemical and mechanical properties, residual solvent/water content, drug release profile, bioactive molecules or drugs dosage, packaging, safety, therapeutic target, patient population, and their acceptability [10].

So, it is urgent to draw attention to the lack of Regulatory Guidelines. These delivery systems are not yet recognized officially because they are not included in Pharmacopoeia (of any country) [8,15]. In general, Pharmacopoeia monographs report the in vitro studies needed to prove the quality of the product. For example, the European Pharmacopoeia/European Directorate for the Quality of Medicines and Healthcare (Ph. Eur./EDQM) reports “Tablets” and the “Oromucosal preparations” monographs, where indications are applicable to the OF [16]. On the other hand, the United States Pharmacopeia/Food and Drug Administration (USP/FDA) reports the definition of OF in the “Pharmaceutical Dosage Forms” monograph. However, there is no uniformity of well-defined criteria and parameters [16,17]. As a strategy to improve the quality of these delivery systems, authors have been reporting “Critical Quality Attributes” [10,11,18]. For example: (i) ensure appropriate mechanical strength (thickness characterization, tensile strength, elongation at break, resistance, endurance, and Young’s modulus), (ii) assure the OF stability (chemical and physical), (iii) assure the microbiological properties, (iv) know the drug’s (active molecules and excipients) release profile and the disintegration processes, (v) ensure the OF organoleptic properties (by sensory analysis), and (vi) ensure the monitoring of OF production [10].

## 3. Advantages of Oral Film Administration

As reported in the literature [6,9,10], OF offers several advantages over other oral formulations (tablets, capsules, drops or syrup formulations), such as:

▪ quickly disintegrating and dissolving in the oral cavity;
▪ fastest onset of action by oral mucosa;
▪ flexible packaging, ensuring ease of transportation and storage;
▪ precision in the administered dose (dose reduction and consequently fewer side effects);
▪ ease of swallowing;
▪ ease of administration, eliminating the need for water upon administration, so it can be consumed at any place and anytime as per the convenience of the individual;
▪ bioactive molecules or drugs can be absorbed directly into the oral cavity or by entering the systemic circulation, thereby avoiding the first-pass hepatic metabolism;
▪ bioavailability (less dosage);
▪ can include several types of drugs or bioactive molecules;
▪ beneficial for dysphagia, psychiatric, children, and older patients;
▪ acceptability for the consumer in terms of convenience (easy administration, appearance, composition, taste, and mouthfeel) and social stigma associated with other oral formulations.

The advantages we have mentioned make these drugs or bioactive molecules delivery systems worthy of consideration for pharmaceutical companies. The incorporation of bioactive molecules acting on the central nervous system has been used in the composition of these delivery systems and has aroused interest in neuroscience.

Despite the advantages of these delivery systems, it should be noted that there are also some disadvantages, the most prominent of which is the low capacity to load the drug.

## 4. Oral Film Application in Mental Disorders

Excessive antipsychotic consumption is a public health problem and innovative strategies must be devised. The common way of consuming psychotic drugs, through injectable and oral administration (tablets, capsules, syrups), has its limitations. Some limitations of antipsychotic drugs include the poor bioavailability (because of the large first-pass metabolism); poor solubility; inability to pass through the blood–brain barrier (BBB) in sufficient amounts to exert their therapeutic effect; low plasma half-life; high metabolic clearance; and significant adverse side effects [19]. On the other hand, oral administration is the most commonly used manner for frequent administrations, but not for emergencies. Injectable administration is preferred for emergencies because of its quick response [19]. One significant disadvantage is the invasiveness of this methodology, which can reduce patient compliance, and requires a qualified person for administration. Therefore, the search for new delivery systems and new bioactive molecules is increasing with the aim of improving performance in terms of drug efficiency and ease of consumption.

OF, an innovative delivery system, seems to be an attractive solution in terms of consumer acceptance, comfort, ease of swallowing, and reduction in cultural stigmatization. The acceptance of medication by these patients is similar to that of children and elderly people, and it is often difficult, both in terms of swallowing and the stigma of taking medication. Pediatric patients, as well as psychiatric patients often have unique requirements and considerations regarding medication acceptance. Factors such as taste, texture, and ease of administration are crucial in determining their willingness to take oral medications, including oral films. Understanding and addressing these specific needs is essential for ensuring successful treatment outcomes in these vulnerable groups. Similarly, the elderly population also faces challenges in medication acceptance. Issues such as polypharmacy, cognitive impairments, and physical limitations can impact their ability to take medications effectively. The formulation and characteristics of oral films need to be carefully considered to accommodate the preferences and abilities of elderly and psychiatric patients, ensuring optimal medication adherence and efficacy. On the other hand, OF is advantageous in terms of the release of the drug or bioactive molecules (bioavailability, safe delivery, etc.). These new and alternative dosage forms can also be personalized for patients’ needs. Again, these delivery systems can encompass nanoparticles, allowing for more targeted delivery through the BBB to ensure an effective therapeutic dose of the drug. Furthermore, psychiatric patients can have swallowing problems that can be caused by psychological disorders, neurological conditions, or other reasons, such as the adverse effects of psychiatric medications. Therefore, this delivery system can improve treatment adherence as well as the patient’s quality of life [16].

The evolution of these delivery systems is constantly occurring, and the incorporation of new drugs and bioactive molecules in mental health/neurosciences fields is our current reality (Table 1). Table 1 highlights not only the drugs or bioactive molecules incorporated in OF and some features of product development, but also clinical studies using this delivery system (such as OF with Buprenorphine, Dexmedetomidine, Diazepam, Flupentixol dihydrochloride, Montelukast, Paroxetine, Sildenafil), although they are still scarce. The information comprises results from a literature search in the Web of Science, Scopus, and PubMed databases between 2018 and 2023.

Molecules such as aripiprazole, an active pharmaceutical ingredient used to treat several mental diseases (schizophrenia, bipolar disorder, autistic disorder, and Tourette’s disorder), are being incorporated into OF formulations, and the results show advantages to dissolution profile [20,21]. According to the prescribed treatments, the OF may bring benefits in terms of effectiveness and acceptance [22]. Other molecules such as fluoxetine, flupentixol dihydrochloride, olanzapine, paroxetine, and risperidone have also been incorporated into OF to be used in treating schizophrenia, depression, anxiety, bipolar disorder, and acute mixed or manic episodes [39,40,43,46]. For example, paroxetine is a selective inhibitor for the reuptake of the neurotransmitter serotonin by the presynaptic receptors used in the treatment of generalized anxiety disorder, depression, and post-traumatic stress disorder, and it is the first anti-depressant used for the treatment of panic disorders. Paroxetine has a bitter taste, low water solubility, and must undergo extensive first-pass metabolism, leading to poor oral bioavailability. Thus, OF has been developed to control these disadvantages. Recently, paroxetine nanosuspension into OF and chitosan/clay/paroxetine composite films were developed and showed that they could be used as an oral drug delivery system to ensure a steady and prolonged paroxetine delivery [44,45].

Buprenorphine is another drug used in OF formulation (sublingual film or tablet) [49,50,51]. This active pharmaceutical ingredient, approved by the Food and Drug Administration, is mainly used to combat opioid use disorder treatment (or opioid withdrawal treatment) and treatments for moderate to severe pain [49,50]. The pharmacokinetic profile of buprenorphine varies considerably between individuals partly because of the drug’s bioavailability [51]. Sometimes buprenorphine is combined with another opioid antagonist, naloxone [52]. These molecules were originally used in OF formulations and have been shown to increase bioavailability while dissolving compared to other products [51,52,53,54]. Sumatriptan succinate is a serotonin receptor agonist which can also be included in OF, and used to treat migraines [48].

OF is also used in epilepsy treatment; for example, diazepam buccal film has shown advantages when compared with other dosage forms, because the buccal film is a convenient dosage form and presents appropriate dosing, allowing a higher performance than others [34]. Likewise, an OF is also being developed for the treatment of major depressive disorder and anxiety disorder. Escitalopram and quetiapine are molecules that can also be included. Escitalopram is a serotonin reuptake inhibitor, increasing the serotonin levels in the synaptic clefts [37]. Quetiapine is an atypical antipsychotic approved by the United States Food and Drug Administration that antagonizes dopamine, adrenergic, histaminergic, and serotonergic receptors [37]. In addition, quetiapine has been used for schizophrenia and bipolar disorder treatment [37].

An OF has also been developed to carry active pharmaceuticals such as donepezil and montelukast for application in patients with Alzheimer’s disease [41,55]. The donepezil OF has demonstrated significant efficacy in maintaining cognitive activities and treating symptoms of Alzheimer’s and dementia. There are clinical trials present in the literature about this molecule, namely the commercial product “Aricept”, but the route of administration, although oral, is not by OF [56,57]. Montelukast (MTK) is a leukotriene receptor antagonist commonly used to treat chronic asthma, but recently it has also been used in the treatment of Alzheimer’s disease. Recent literature has highlighted the power of this substance to reduce the risk of dementia and to improve cognitive function in patients with dementia [42]. An MTK OF could therefore be used in the future as a therapeutic product in acute and chronic neurodegenerative diseases.

The industry is investing in this modality of oral devices, although other forms of oral administration are more commonly used, such as tablets, particularly orodispersible tablets, or film-coated tablets [58,59]. Nonetheless, OFs have advantages, such as those mentioned above, and recently the FDA in 2022 approved the sublingual formulation of dexmedetomidine (Igalmi) as a novel therapeutic for the treatment of acute agitation associated with schizophrenia or bipolar I or II disorder in adults [27,28].

These are some recent examples that show OF’s application to mental disorders. There are other neuromodulator substances that can be included in OFs that impact the central nervous system with therapeutic applications for pain, migraine, sleep disorders, and/or stimulating functions [15]. However, the focus of this manuscript is to draw attention to mental disorders and the importance of the use of films in terms of product acceptance and effectiveness.

## 5. Oral Film in Mental Health: S.W.O.T. Analysis

**Figure 2:**
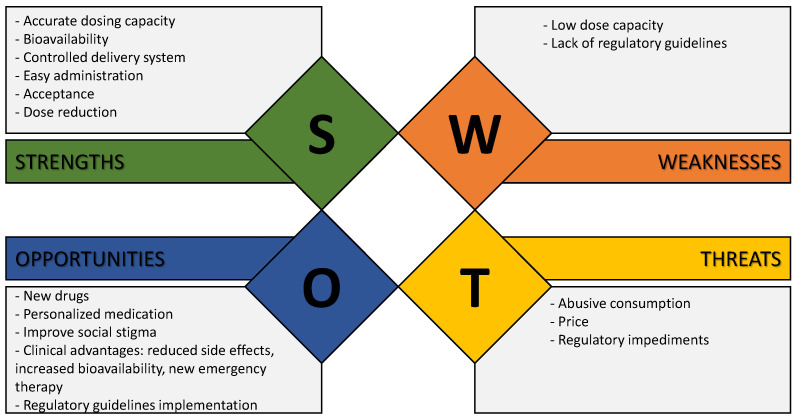
S.W.O.T. analysis for OF application in mental health.

The application of oral films in the field of mental health and neurosciences is evolving in terms of scientific research, arousing the interest of the pharmaceutical industry. Reflecting on this theme is important, which garners the necessity of a S.W.O.T. analysis (strengths–weaknesses–opportunities–threats). This analysis allows product analysis for subsequent implementation in industry and society (see Figure 2).

### 5.1. Strengths

There is increased research in biotechnology and neuroscience searching for drugs and bioactive molecules to prevent and treat mental disorders. Additionally, the search for natural molecules promotes the sustainability of the products formed.

The development of controlled release systems, which is easy to handle and use and allows the incorporation of a specific dose (a characteristic especially relevant for those drugs that have a very narrow therapeutic range) [11] is increasingly desired. Additionally, it does not require high doses (unlike other forms of drug delivery), ensuring that the specific dose is effective because it is possible to increase the bioavailability in the body since it avoids the first-pass metabolism that occurs when ingested orally. These devices still have a strong consumer acceptance due to their easy and comfortable administration, and are highly versatile since it is possible to formulate a product to deliver a wide range of drugs [11].

### 5.2. Weaknesses

The most significant obstacle for the OF is that it cannot be used for higher dosages of active pharmaceutical ingredients (or natural bioactive molecules) since OF may not be suitable for drugs that require high doses because of its drug loading. OFs usually do not exceed 25 mg/film, as dosages of this size would compromise the physical and chemical characteristics of the products. Additionally, a balance between the comfort of the product at the level of consumption (texture, hardness) and its fast disintegration must be achieved. Other challenges include ensuring the insolubility of the drug(s) or bioactive molecule(s), taste masking, and maintaining the stability of OF against humidity and temperature. On the other hand, the manufacturing process is more expensive than other traditionally administration forms.

Another weakness is a lack of regulation of OF production to ensure quality and safety [12].

### 5.3. Opportunities

There are great opportunities for the pharmaceutical and nutraceutical industry. These new delivery systems allow one to control the dosage, easily meeting more specific criteria for a particular type of patient and indicating the move towards personalized medication.

The number of consumers (especially target audiences) is believed to increase with accessibility and product knowledge. Consequently, the treatment adhesion will be beneficial.

Another big opportunity is the reduction in side effects of the medication and improvement of consumer acceptance. In particular, combatting social stigmatization (so frequent in mental disorders patients) represents an opportunity for both patients and health professionals. Another clinical advantage is using this OF in drug therapies where a fast onset action is essential. Finally, the implementation of regulatory guidelines will allow the monitoring of the production of the products as well as the guarantee of their quality and effectiveness.

### 5.4. Threats

Although oral films have several advantages as a drug delivery system, some potential threats and limitations should be considered. One is the manufacturing process which requires specialized equipment, especially for large-scale production. This problem impacts on unprofitable sales and the industry’s preference for producing and marketing traditional forms of administration, such as tablets or capsules.

Quality control can be challenging due to the delicate nature of the OF and the intolerance regarding variations in the compositions. The manufacturing process is critical to avoid any safety concerns, for instance, if the OF will dissolve correctly.

The biggest threat to production is the lack of certain general regulations. Another equally important fear is the widespread consumption of these products, namely nutraceutical oral films. It is still important not to forget that the product price may also condition its adhesion.

## 6. Conclusions

Mental disorders are increasing, so new strategies and products, as well as higher consumer literacy, are needed. New products and innovative delivery systems for drug or bioactive molecule release, with increased efficiency and acceptance by consumers, can be an innovative strategy that could reduce these pathologies or even prevent their appearance.

This review intended to draw attention to these new delivery systems that use a promising technology and have been used for the incorporation of molecules into the nervous system, among other systems. However, the lack of regulatory guidelines is a limiting condition, and it is important to reflect and act on this point. More research is required to establish global standards to ensure the quality and safety of these products. Likewise, we draw attention to the pertinence and relevance of its potential for application in mental disorders.

## Figures and Tables

**Table 1:** Oral film development and clinical trials for mental disorders treatment.

Drug or Bioactive Molecule	Therapeutic Application	Commercial Product	Product Development		Clinical Trials			References
			Technological Process	Polymers	Participants	Type of Study/Stage	Dosage	
Aripiprazole	- Schizophrenia - Bipolar mania - Autistic disorder - Tourette disorder	-	Solvent casting method	PVA	-	-	-	[20,21]
		-	3D printing	PVA	-	-	-	[22]
Buprenorphine/ Naloxone	- Opioid dependence	Buccal Film Formulation Buprenorphine-naloxone (BEMA)	BEMA technology	-	249 subjects	Open-label study	3.5/0.6 mg and 5.25/0.9 mg	[23]
		Suboxone	-	-	566 patients	Prospective, randomized, parallel-group	8/2 or 2/0.5 mg	[24]
		Suboxone (Indivior, Slough, UK)		-	350 subjects	Open-label, randomized controlled, comparative effectiveness trial	4 mg/1 mg and 8 mg/2 mg strengths.	[25]
		Suboxone	-	-	24 patients	Observational retrospective case series study amongst an opioid-use disorder patient population of a single clinic	-	[26]
Dexmedetomidine	- Schizophrenia - Bipolar disorders	-	-	-	135 patients (Phase 1) 381 patients (Phase 3)	Review: SERENITY 1 pivotal and SERENITY 2 pivotal trial Phase 1 and 3	180 and 120 μg	[27]
		Igalmi	-	-	759 patients	phase 3 randomized, double-blind, placebo-controlled trials	180 and 120 mg	[28]
	- Schizophrenia or schizoaffective disorder	-	-	-	380 patients	Phase 3, randomized, double-blind, placebo-controlled study	80 and 120 mg	[29]
	- Bipolar disorders	Sublingual dexmedetomidine (BXCL501, BioXcel Therapeutics)	-	-	380 patients	Phase 3, randomized, double-blind, placebo-controlled trial	90 and 60 μg	[30]
Diazepam	- Epilepsy	-	-	-	36 heathy subjects	Single-dose, randomized, 4-period, 4-sequence, open-label crossover at a single site	5 and 15 mg	[31,32]
		-	-	-	118 patients	phase 3, multicenter, open-label, long-term safety and tolerability study	5–17.5 mg	[33]
	- Epilepsy (acute seizures)	Buccal soluble film formulation of diazepam (DBF; Libervant™, Aquestive Therapeutics)	-	-	35 patients	Single-dose, randomized, open-label, three-period,	5, 10 and 15 mg	[34]
Donepezil	- Alzheimer’s disease	-	Solvent casting method	Polyetheylene	-	-	5 mg	[35]
		-	Solvent casting method	HPMC	-	-	23 mg	[36]
Escitalopram/ Quetiapine	- Major depressive disorder	-	Electrospinning	Polyvinylpyrrolidone (PVP)	-	-	-	[37]
Fluoxetine	- Major depressive disorder	-	Solvent casting method	PVA	-	-	-	[38]
	- Psychotic disorders of selective mutism - Obsessive compulsive disorder	-	Solvent casting evaporation method	Hydroxypropyl methylcellulose (HPMC)	-	-	-	[39]
Flupentixol dihydrochloride	- Schizophrenia - Depression - Anxiety	-	Solvent casting method	HPMC	6 heathy subjects	Blind, two-treatment parallel design	1 mg	[40]
Montelukast	- Alzheimer’s disease	Montelukast VersaFilmTM	-	-	-	Phase II clinical study	-	[41]
		-	Solvent casting method		8 heathy subjects	Phase I clinical study	10 mg	[42]
Olanzapin	- Schizophrenia - Acute mixed or manic episodes - Bipolar disorder	-	Solvent casting method	HPMC	-	-	-	[43]
Paroxetine	- Generalized anxiety disorder - Depression - Post-traumatic stress disorder - Panic disorders	-	Solvent casting method	Chitosan/clay/paroxetine	-	-	-	[44]
		-	Solvent casting method	Carboxymethyl cellulose (CMC)	8 heathy subjects	Double-blind crossover study	25 mg	[45]
Risperidone	- Schizophrenia	-	Solvent casting method	HPMC	-	-	-	[46]
Sildenafil	- Multiple sclerosis - Parkinson’s disease - Depression - Traumatic stress disorders thers	Sildenafil OF developed by IBSA is approved in Europe	-	-	53 heathy subjects	Single-center, single-dose, randomized, open, two-way crossover study	100 mg	[47]
Sumatriptan succinate	- Migraine	-	Solvent casting method	PVA or HPMC	-	-	-	[48]

(-) information not applicable or unavailable.
